# Mediastinal parathyroid cyst: A case report and review of the literature

**DOI:** 10.1016/j.radcr.2024.06.049

**Published:** 2024-07-06

**Authors:** Fahmi H. Kakamad, Abdulwahid M. Salih, Aras J. Qaradakhy, Ari M. Abdullah, Hezha A. Mohammed, Rebaz O. Mohammed, Hiwa O. Baba, Shaho F. Ahmed, Shko H. Hassan, Marwan N. Hassan, Abdullah A. Qadir

**Affiliations:** a Smart Health Tower, Sulaymaniyah, Kurdistan, Iraq; b College of Medicine, University of Sulaimani, Sulaymaniyah, Kurdistan, Iraq; c Kscien Organization, Sulaymaniyah, Kurdistan, Iraq; d Department of Radiology, Shorsh Teaching Hospital, Sulaymaniyah, Kurdistan, Iraq; e Department of Pathology, Sulaymaniyah Teaching Hospital, Sulaymaniyah, Kurdistan, Iraq

**Keywords:** Parathyroid gland, Mediastinal cyst, Hyperparathyroidism, Video-assisted thoracoscopic surgery

## Abstract

Mediastinal parathyroid cysts (MPCs) are extremely rare, benign lesions arising from the parathyroid glands and residing within the thoracic cavity. This study aims to advance understanding of MPC, emphasizing accurate diagnosis and management approaches for this rare condition. A 46-year-old woman presented with dysphagia for one week. Blood tests revealed elevated parathyroid hormone (PTH) (112.8 pg/mL) and normal serum calcium (9.54 mg/dL). Ultrasonography identified a large, well-defined cystic nodule measuring 46 × 30 × 25 mm, extending retro-sternally in the right upper third of the chest. A subsequent high-resolution computed tomography scan of the chest revealed a large space-occupying lesion (47 × 43 × 31 mm) in the superior mediastinum, near the esophagus, suggesting an esophageal duplication cyst or, less likely, a bronchogenic cyst. Video-assisted thoracoscopic surgery (VATS) was performed, and the entire cyst was excised, confirmed histologically as a mediastinal parathyroid cyst. Mediastinal involvement of PCs poses diagnostic challenges due to their rarity and diverse clinical presentations. Surgical excision is necessary for symptomatic cases, with VATS emerging as a favorable approach.

## Background

Parathyroid cysts (PCs) are benign lesions that are extremely uncommon, accounting for only 0.075% of parathyroid gland pathologies [1]. These cysts usually arise in the neck [2]. Although they are mostly nonfunctional and asymptomatic, PCs can be functional, causing hyperparathyroidism symptoms [3,4]. The exact pathogenesis of PC is not entirely understood [5]. They can affect individuals of any age, with a peak incidence between 40 and 60 years old, with no gender predilection [3]. Despite the potential for diverse clinical presentations, most mediastinal cysts remain asymptomatic, leading to infrequent preoperative diagnoses. The subtle nature of these cysts presents a significant diagnostic challenge in distinguishing them from other mediastinal entities like bronchogenic cysts, duplication cysts, and even thymus cysts [6].

This study aims to contribute to the understanding of mediastinal parathyroid cysts (MPCs), emphasizing the importance of accurate diagnosis and appropriate management strategies for this rare condition.

## Case presentation

Patient information: A 46-year-old female patient presented with difficulty swallowing for 1-week duration. Patients past medical history was unremarkable.

Clinical finding: Vital signs were stable on examination. There was unremarkable neck examination. Physical examinations of the chest, abdomen, skin, and extremities were unremarkable.

Diagnostic approach: The thyroid stimulating hormone (TSH) test was within the normal range (2.46 mIU/mL), the serum calcium level was 9.54 mg/dL, and the parathyroid hormone (PTH) was 112.8 pg/mL.

**Fig. 1:**
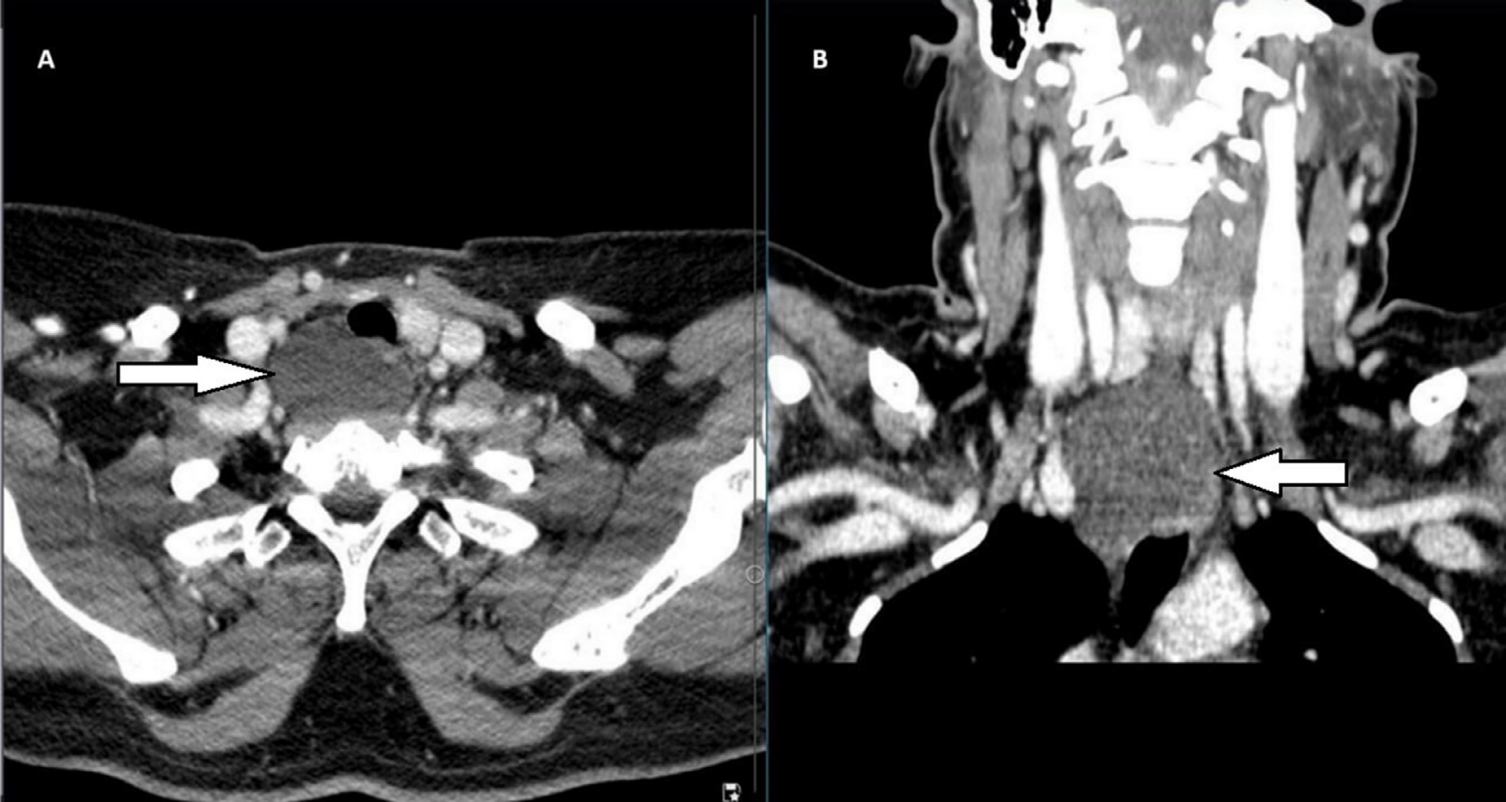
CT scan of neck and upper chest with contrast (axial and coronal section), shows well defined thin wall cystic lesion (white arrow), located in right side posteriolateral to trachea and extending to the mediastinum, causing mild surrounding mass effect, with no enhancement.

Neck ultrasound: The neck ultrasound (US) revealed a large cystic nodule with a well-defined, regular surface measuring 46 × 30 × 25 mm, located in the posterior aspect of the base of the neck. On the chest computer tomography (CT) scan, a large fluid-filled cystic lesion was evident, measuring 47 × 43 × 31 mm, located in the superior mediastinum. It was adjacent to the right lateral wall of the esophagus without obvious communication to the esophagus, neither to the trachea nor to the thyroid gland, suspecting an esophageal duplication cyst (Fig. 1). The patient was sent for esophago-gastro-duodenoscopy (OGD) because of the differential diagnosis of the esophageal duplication cyst, and the result of the OGD was normal.

Therapeutic intervention: Under general anesthesia, the patient was positioned in the left lateral position for a single port video-assisted thoracoscopic surgery (VATS). The mass was inoculated, hemostasis was achieved, a chest tube was inserted, and the wound was closed in layers. After surgery, the tissue was sent for histopathological examination (HPE). There were no complications following surgery, and the patient's vital signs were stable. The patient exhibited symptomatic improvement postoperatively.

**Fig. 2:**
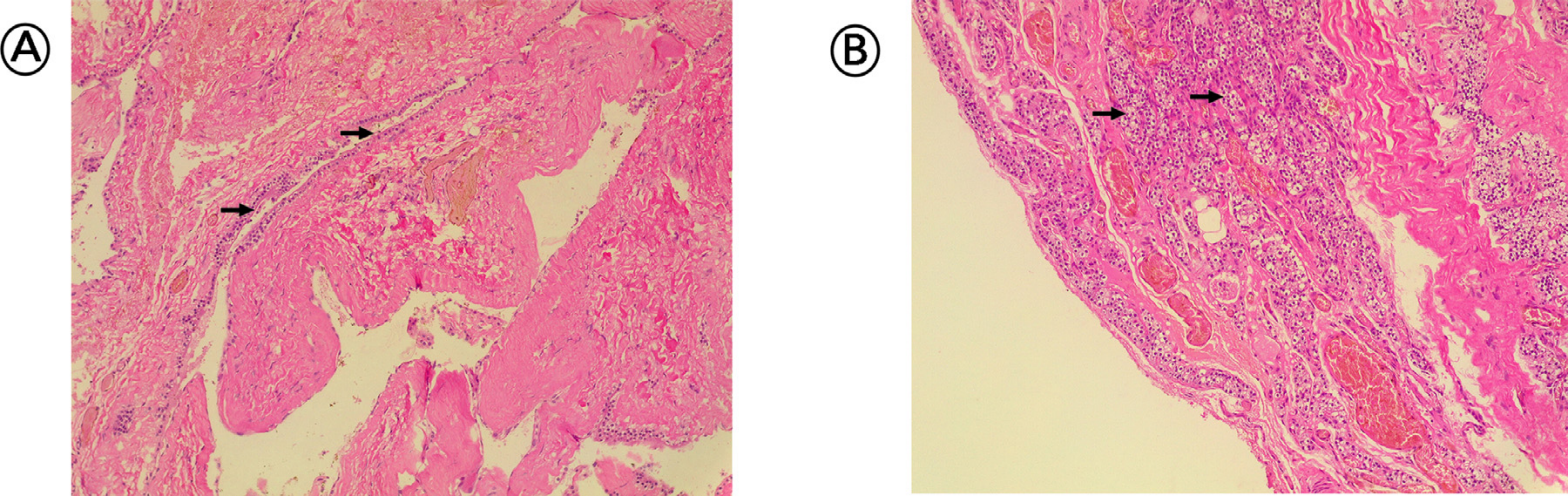
(A) wall of the cyst containing nests of parathyroid gland cells (dark arrows), (B) wall of the cyst that is lined by parathyroid cells (dark arrows).

Histopathological analysis of parathyroid cyst: Histopathological findings of the parathyroid cyst are presented in Fig. 2. In panel A, the cyst wall is depicted, containing nests of parathyroid gland cells, highlighted by dark arrows. Panel B shows the lining of the cyst, which is composed of parathyroid cells. These findings are indicative of a parathyroid cyst and help rule out malignancy.

Follow-up and outcome: Postoperatively, the patient recovered well without complications. Three weeks after surgery, blood calcium levels decreased to 9.14 mg/mL, and PTH levels dropped to 62 pg/dL.

## Discussion

Parathyroid cysts are rare conditions, and even rarely located in the mediastinum [7]. First described in 1918 by Swedish anatomist Sandstrom, they usually arise from the inferior parathyroid glands but can be located in the mediastinum [[7], [8], [9]]. There are several theories for the pathogenesis of these cysts, including remnants of branchial clefts, retention cysts, coalescence of numerous minute cystic lesions, or degeneration of an adenoma [10].

Mediastinal parathyroid cyst is a rare type of PC located in the mediastinum and was first described in 1925 by De Quatrain [5]. Ranging from 0.5 to 12 cm, their descent into the mediastinum is attributed to factors like weight and negative intra-thoracic pressure [11]. Functionally, mediastinal parathyroid cysts (MPCs) fall into 2 categories. Functional MPCs cause hyperparathyroidism symptoms. Nonfunctional MPCs are often asymptomatic or present with compression symptoms [12]. Even though functional MPCs have a similar gender distribution, nonfunctional cysts are more frequent in females. Shields et al. found PCs in three areas of the mediastinum in 93 patients; anterior-superior (pretracheal) is the most common site (58.3%), followed by the retro-tracheal area of the visceral compartment (28.1%), and the true anterior or perivascular compartment (13.5%) [13]. The current case presents a female with an antero-superior (pretracheal) MPC-caused dysphagia.

According to Oyama et al., a normal blood calcium level suggests a nonfunctioning PC [14]. A patient with functional MPC presented with an acute hypercalcemic crisis with a blood calcium level of 18.7 mg/dL, as documented by Garbuz et al. [11]. It's worthwhile to mention that there were no signs or symptoms of hypercalcemia in the present case. Furthermore, the current patient showed a high level of PTH and a normal serum calcium level.

Imaging techniques such as US and magnetic resonance imaging (MRI) can be utilized to localize the lesion within the mediastinum. The neck US scan is important for diagnosing PCs because it reveals the cystic nature of the mass and its size. CT and MRI scans have nonspecific findings, but they can easily reveal a homogeneous region as cystic. They are valuable diagnostic tools, particularly when vocal cord paralysis symptoms are present or if the cyst is mediastinal [3]. In the present case, a large cystic nodule in the US of the neck exhibited a well-defined, regular surface with a thin wall, and a CT scan characterized cystic structure in the superior mediastinum. Yang et al. reported a case with a chest CT showing a mediastinal cystic tumor. They misdiagnosed it as a bronchogenic cyst [1]. In the present study, the patient was initially diagnosed as having an esophageal duplication cyst based on CT findings. However, subsequent OGDs excluded this diagnosis.

After reviewing the literature, it was discovered that it is better to avoid fine needle aspiration (FNA) and biopsy in cases of MPCs for 2 reasons: first, the biopsy may not contain a homogenous layer of parathyroid cells, making FNA useless for diagnosis; second, there is a high risk of cyst rupture into the pleural space with cellular dissemination in cases of malignant mediastinum [9]. In the current study, FNA was not performed for the patient.

The definitive diagnosis of MPCs demands histological confirmation through tissue analysis after surgical excision [12]. Surgical excision is advised for functional or nonfunctional MPCs that cause compressive symptoms. MPCs, particularly those greater than 4 cm in size and linked with symptoms such as dysphagia and dyspnea, may necessitate surgical intervention. The surgical method used is determined by the location and size of the cyst. When a PC expands into the mediastinum, around twenty percent of individuals can have it resected via a cervical approach. Historically, sternotomy and thoracotomy were the primary techniques for treating intra-thoracic PC. However, in recent years, thoracoscopic surgery has emerged as an accessible and increasingly used method with remarkable results [2,9]. VATs were used to surgically inoculate the cyst due to compression symptoms in the present case. Following the inoculation of the mediastinal cyst, an official diagnosis of MPC was made.

## Conclusion

The MPC poses diagnostic challenges due to their rarity and diverse clinical presentations. Surgical excision is necessary for symptomatic cases, with VATS emerging as a favorable approach.

## Consent for publication

Not applicable.

## Availability of data and material

All data and materials are kept by the first and corresponding authors.

## Patient consent

Consent has been obtained from the patient and the family of the patient.
